# 单孔与多孔胸腔镜肺叶切除术后3个月患者生活质量比较

**DOI:** 10.3779/j.issn.1009-3419.2023.102.42

**Published:** 2023-11-20

**Authors:** Qi ZHANG, Wei DAI, Xing WEI, Run XIANG, Hang GU, Peihong HU, Mingxin LIU, Wei CHEN, Huaijun GONG, Yong LIANG, Shichao ZHANG, Weixing PENG, Qiuling SHI, Qiang LI, Nanbin YU

**Affiliations:** ^1^643020 自贡，自贡市第三人民医院胸外科; ^1^Department of Thoracic Surgery, Zigong Third People's Hospital, Zigong 643020, China; ^2^610041 成都，四川省肿瘤医院·研究所，四川省肿瘤临床医学研究中心，四川省癌症防治中心，电子科技大学附属肿瘤医院胸外科; ^2^Department of Thoracic Surgery, Sichuan Clinical Research Center for Cancer, Sichuan Cancer Hospital & Institute, Sichuan Cancer Center, Affiliated Cancer Hospital of University of Electronic Science and Technology of China, Chengdu 610041, China

**Keywords:** 肺肿瘤, 单孔胸腔镜, 多孔胸腔镜, 生活质量, 患者报告结局, Lung neoplasms, Uniportal thoracoscopy, Multiportal thoracoscopy, Quality of life, Patient-reported outcomes

## Abstract

**背景与目的:**

肺癌术后3个月的生活质量在不同手术入路之间的关系尚不明确，本研究旨在对比单孔与多孔胸腔镜肺叶切除术后3个月患者的生活质量。

**方法:**

收集2021年4月至2021年10月在四川省肿瘤医院胸外科行肺部手术患者的资料，采用欧洲癌症研究与治疗组织生活质量核心量表C30（European Organization for Research and Treatment of Cancer quality of life core 30, EORTC QLQ-C30）和肺癌生活质量量表29（Quality of Life Questionnaire-Lung Cancer 29, QLQ-LC29）收集患者的生活质量资料。将基线资料中潜在混杂因素纳入多因素回归模型中校正，比较两组患者术后3个月的生活质量与传统临床结局。

**结果:**

共纳入130例肺癌患者，男性57例（43.8%），女性73例（56.2%），平均年龄（57.1±9.5）岁。两组患者基线资料中，放置引流管数量具有统计学差异（P<0.001）。经回归模型校正后，在术后3个月时，两组患者全部症状及功能状态评分无明显差异（P均>0.05）。多孔组的手术时间（120.0 min vs 85.0 min, P=0.001）、术后住院时间（6.0 d vs 4.0 d, P=0.020）比单孔组更长，早期≥2级并发症发生率（39.0% vs 10.1%, P=0.011）比单孔组更高。

**结论:**

单孔与多孔胸腔镜肺叶切除术患者在术后3个月时具有相似的生活质量。单孔组在手术时间、术后住院时间及术后早期并发症等传统临床结局指标上可能具有一定的优势。

## Abstract (en)

**Background and objective:**

The relationship between quality of life at three months after lung cancer surgery and different surgical approaches is remains unclear. This study aimed to compare the quality of life of patients three months after uniportal and multiportal thoracoscopic lobectomy.

**Methods:**

Data from patients who underwent lung surgery at the Department of Thoracic Surgery, Sichuan Cancer Hospital between April 2021 and October 2021 were collected. The European Organization for Research and Treatment of Quality of Life Questionnaire-Core 30 (EORTC QLQ-C30) and Quality of Life Questionnaire-Lung Cancer 29 (EORTC QLQ-LC29) were used to collect quality of life data of the patients. Potential confounding factors in the baseline data were included in a multivariate regression model for adjustment, and the quality of life of the two groups three months postoperatively was compared with traditional clinical outcomes.

**Results:**

A total of 130 lung cancer patients were included, with 57 males (43.8%) and 73 females (56.2%), and an average age of (57.1±9.5) yr. In the baseline data of the two groups, there was a statistical difference in the number of chest drainage tubes placed (P<0.001). After adjustment with the regression model, at three months postoperatively, there were no significant differences in all symptoms and functional status scores between the two groups (all P>0.05). The multiportal group had longer surgery time (120.0 min vs 85.0 min, P=0.001), postoperative hospital stay (6.0 d vs 4.0 d, P=0.020), and a higher incidence of early ≥ grade 2 complications (39.0% vs 10.1%, P=0.011) compared to the uniportal group.

**Conclusion:**

Patients undergoing uniportal and multiportal thoracoscopic lobectomy have similar quality of life at three months postoperatively. The uniportal group may have certain advantages in terms of traditional clinical outcome indicators such as operation time, postoperative hospital stay, and early postoperative complications.

肺癌是我国发病率和死亡率最高的恶性肿瘤之一^[1]^。随着低剂量计算机断层扫描（computed tomography, CT）在健康筛查中的运用，早期肺癌患者的检出率逐渐提高^[2]^。手术是早期肺癌的重要治疗手段，但患者在术后存在较多的症状^[3]^。随着快速康复理念的发展^[4]^，手术时间、术后住院时间、出血量、围手术期并发症、生存时间等传统临床指标存在一定的局限性，其未能反映患者的症状功能及生活质量，而患者报告结局（patient-reported outcomes, PROs）能很好地弥补这一缺失^[5]^，PROs是指直接来自于患者，未经他人解释的反映其自身健康相关的生活质量、症状负担和功能状态的测量报告。有研究^[6⇓-8]^表明，胸腔镜较传统开胸术后患者的生活质量更好，但传统胸腔镜手术通常在多操作孔下进行，随着肺癌外科技术的发展，微创入路方式逐步由多孔转向为单孔胸腔镜。既往的研究表明，单孔比多孔在出血量、手术时间方面具有一定的优势^[9,10]^，单孔胸腔镜手术有望成为肺癌外科标准术式之一^[11]^。但单孔胸腔镜和多孔胸腔镜肺癌患者术后生活质量的相关研究有限，而患者术后生活质量有必要得到关注^[12]^。

肺癌患者术后生活质量的测量需要特定的工具。既往肺癌PROs测量工具并不是针对肺癌手术患者的特异性工具^[13⇓-15]^。欧洲癌症研究与治疗组织（European Organization for Research and Treatment of Cancer, EORTC）近期将肺癌生活质量量表13（Quality of Life Questionnaire-Lung Cancer 13, QLQ-LC13）更新为肺癌生活质量量表29（QLQ-LC29），新量表首次增加了评估肺癌患者手术相关症状的特异性条目。新量表仍需与癌症患者生活质量核心量表（Quality of Life core 30, QLQ-C30）共同使用，其中5条是专用于肺癌手术患者症状评估的特异性条目^[16,17]^。有研究^[17]^发现该量表在国外肺癌患者中具有良好的心理测量学属性，我们团队完成了QLQ-LC29的汉化工作^[18]^和信效度检验工作^[19]^。然而，目前在国内尚缺乏应用QLQ-LC29比较单孔和多孔胸腔镜肺癌术后的研究。本研究主要旨在应用QLQ-LC29这一新型肺癌特异性生活质量测量工具，比较单孔和多孔胸腔镜肺叶切除术后3个月患者的生活质量。

## 1 资料与方法

### 1.1 患者资料

基于一项在四川省肿瘤医院进行的前瞻性、观察性纵向队列研究（CN-PRO-Lung 4）的数据进行回顾性分析。图1为患者的筛选过程。具体纳入标准为：（1）年龄≥18岁；（2）行胸腔镜肺叶切除术；（3）无其他恶性肿瘤史。排除标准为：（1）术前新辅助治疗；（2）术后行后续治疗；（3）接受二次手术；（4）术后病理诊断不是非小细胞肺癌；（5）术前和术后3个月PROs资料收集不完整。将所纳入的患者分为单孔组和多孔组，其中多孔组包括两孔、三孔及四孔胸腔镜。收集患者的一般资料、手术资料、病理资料和并发症等临床资料，收集术前及术后3个月的PROs数据。

**图1:**
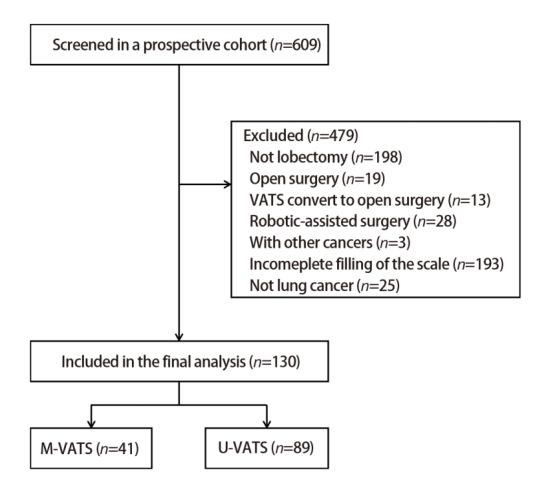
患者筛选过程

### 1.2 手术方式及术后管理

患者均在全身麻醉、双腔气管插管、侧卧位、单肺通气的条件下手术。单孔胸腔镜组手术仅有1个位于患侧第4或5肋间腋前线与腋中线之间的切口，长度为3-5 cm^[13]^。置入切口保护套，使用30°腔镜与腔镜下直线切割吻合器完成肺叶切除术及淋巴结清扫术，手术完成后于切口边缘留置胸腔闭式引流管。对于多孔组则有2-4个直径在0.5-4 cm之间的切口，30°腔镜从操作孔以外的切口辅助操作完成肺叶切除术及淋巴结清扫术，术毕留置胸腔闭式引流管。

术后按胸外科常规护理，鼓励患者咳嗽排痰并早期下床活动，术后复查胸片提示肺复张良好、引流管无漏气、无乳糜胸、无出血且引流量≤200 mL/d时拔除引流管。

### 1.3 生活质量测量工具及测量时间点

患者生活质量的评估使用QLQ-C30和QLQ-LC29（信效度检验结果示：量表的克朗巴赫系数均>0.7，拟合指数>0.85）作为测量工具。QLQ-LC29共29个条目，包括咳嗽、气短、治疗副反应、害怕疾病进展和手术相关症状等5个多条目症状，咯血、胸痛、肩手痛、体重下降和身体其他部位疼痛等5个单条目症状。每个条目的评分为1-4分，分别代表“没有”“有点”“相当”“非常”四个等级。多条目的得分计算方法为各条目数评分之和除以条目数即得到粗得分，最终按线性转换规则转换为0-100分即为最终得分。最终得分越高表示该症状越严重。PROs数据采集时间节点为术前及术后3个月，数据采集方式主要通过电子版问卷进行，纸质版问卷和电话随访作为补充。

### 1.4 结局指标

本研究主要结局指标是患者术后3个月的症状、功能及生活质量。次要结局指标是传统临床结局，包括手术时间、出血量、住院时间、术后住院时间和术后并发症发生率（术后3个月）。并发症的收集采用的是Clavien-Dindo并发症分级系统，测量2级及以上的并发症发生率。

### 1.5 统计分析

对部分条目回答缺失的患者进行了得分项调整，剔除了15项及以上条目缺失的患者^[16]^。计量资料用均数±标准差（Mean±SD）或中位数及四分位数间距表示，分类变量用例数及百分比表示。采用t检验、Mann-Whitney U检验、卡方检验和Wilcoxon配对检验进行统计分析。将基线资料中放置引流管数量纳入回归模型中对生活质量结局指标进行校正，比较两组患者术后3个月的生活质量。使用SPSS 26.0软件进行数据分析，P<0.05被认为差异有统计学意义。

### 1.6 伦理审查

本研究已通过四川省肿瘤医院伦理委员会批准，审批号：SCCHEC-02-2020-066。本研究在中国临床试验注册中心的注册号：ChiCTR2000041514。

## 2 结果

### 2.1 患者一般特征

表1为患者人口统计学特征。CN-PRO-Lung 4共纳入了609例肺切除术患者，本研究排除了479例不符合纳入标准的患者，共有130例患者被纳入分析。其中单孔组41例，多孔组89例，两组患者平均年龄（57.1±9.5）岁。两组患者在基线资料中放置引流管数量存在统计学差异（P<0.001）。两组患者在年龄、体质指数、性别、民族、受教育程度、美国东部合作肿瘤小组评分（Eastern Cooperative Oncology Group, ECOG）、美国麻醉医师学会分级（American Society of Anesthesiologists, ASA）、查尔森合并症指数（Charlson Comorbidity Index, CCI）、肺功能、吸烟史、术后病理类型及病理分期等方面差异均无统计学意义（P均>0.05）。

**表1:** 患者临床特征

Variables	U-VATS (n=89)	M-VATS (n=41)	P
Age, yr, Mean±SD	58.0±9.9	57.3±8.3	0.143
Gender, n (%)			0.711
Male	40 (44.9)	17 (41.5)	
Female	49 (55.1)	24 (58.5)	
BMI, kg/m^2^, Mean±SD	23.0±3.0	23.0±2.3	0.907
Education level			0.116
Below middle school graduate	41 (46.1)	25 (61.0)	
Middle school graduate or above	48 (53.9)	16 (39.0)	
ECOG score, n (%)			0.946
0	87 (97.8)	40 (97.6)	
≥1	2 (2.2)	1 (2.4)	
ASA classification, n (%)			0.421
1	87 (97.8)	39 (95.1)	
>1	2 (2.2)	2 (4.9)	
CCI score, n (%)			0.979
0	78 (87.6)	36 (87.8)	
≥1	11 (12.4)	5 (12.2)	
FEV_1_, n (%)			0.096
<1.5 L	25 (28.1)	6 (14.6)	
≥1.5 L	64 (71.9)	35 (85.4)	
Smoking status, n (%)			0.812
Never smoked	69 (77.5)	31 (75.6)	
Current smoker	12 (13.5)	6 (14.6)	
Former smoker	8 (9.0)	4 (9.8)	
Thoracic drainage tube, n (%)			<0.001
1	74 (83.1)	12 (23.9)	
2	15 (16.9)	29 (70.7)	
Histological type, n (%)			0.236
Adenocarcinoma	86 (96.6)	41 (100.0)	
Squamous cell carcinoma	3 (3.4)	0 (0.0)	
pTNM stage (8^th^ edition), n (%)			0.244
0-I	68 (76.4)	35 (85.4)	
≥II	21 (23.6)	6 (14.6)	

BMI: body mass index; ECOG: Eastern Cooperative Oncology Group; ASA: American Society of Anesthesiologists; CCI: Charlson comorbidity index; FEV_1_: forced expiratory volume in one second; pTNM: pathological tumor-node-metastasis.

### 2.2 PROs指标

表2为全部患者术前与术后3个月生活质量的比较。在术后3个月时，患者的生活质量有不同程度的下降，主要表现为咳嗽（P=0.003）、气短（P<0.001）、害怕疾病进展（P<0.001）、咳血（P<0.001）、胸痛（P=0.002）和体重下降（P=0.020）较术前严重。

**表2:** 所有患者术前和术后生活质量

Variables	Preoperative	Postoperative	P
Coughing, median (IQR)	16.7 (0.0-33.3)	33.3 (0.0-33.3)	0.003
Shortness of breath, median (IQR)	5.6 (0.0-11.1)	22.3 (11.0-33.3)	<0.001
Side effects of treatment, median (IQR)	5.6 (0.0-11.1)	5.7 (2.7-11.0)	0.458
Fear of progression, median (IQR)	33.3 (29.2-50.0)	33.3 (0.0-33.3)	<0.001
Coughing blood/haemoptysis, median (IQR)	0.0 (0.0-0.0)	33.3 (0.0-33.3)	<0.001
Pain in chest, median (IQR)	0.0 (0.0-33.3)	0.0 (0.0-33.3)	0.002
Pain in arm or shoulder, median (IQR)	0.0 (0.0-33.3)	0.0 (0.0-33.3)	0.795
Pain in other parts of body, median (IQR)	0.0 (0.0-33.3)	0.0 (0.0-33.3)	0.330
Weight loss, median (IQR)	0.0 (0.0-33.3)	0.0 (0.0-0.0)	0.020

IQR: interquartile range.

表3为两组患者术前及术后3个月生活质量的比较。在术前基线资料中，两组患者的咳嗽、气短、治疗副反应、咳血、胸痛、肩手痛及体重下降无统计学差异（P均>0.05），害怕疾病进展（P=0.007）和其他部位疼痛（P=0.022）有差异。在术后3个月时，两组的咳嗽、气短、治疗副反应、害怕疾病进展、手术相关症状、咯血、胸痛、肩手痛、其他部位疼痛和体重下降无统计学差异（P均>0.05），两组患者的躯体功能、角色功能、情绪功能、认知功能、社会功能、总健康状况、乏力、恶心与呕吐、疼痛、气短、失眠、食欲丧失、便秘、腹泻和经济困难无统计学差异（P均>0.05）。

**表3:** 单孔组与多孔组患者生活质量比较

Variables	U-VATS (n=89)	M-VATS (n=41)	P
Preoperative PROs, median (IQR)			
Coughing	16.7 (0.0-33.3)	16.7 (0.0-33.3)	0.438
Shortness of breath	0.0 (0.0-11.1)	0.0 (0.0-11.1)	0.623
Side effects of treatment	5.6 (0.0-11.1)	5.6 (0.0-11.1)	0.744
Fear of progression	33.3 (33.3-66.7)	33.3 (33.3-66.7)	0.007
Coughing blood/Haemoptysis	0.0 (0.0-0.0)	0.0 (0.0-0.0)	0.121
Pain in chest	0.0 (0.0-0.0)	0.0 (0.0-33.3)	0.270
Pain in arm or shoulder	0.0 (0.0-33.3)	0.0 (0.0-33.3)	0.197
Pain in other parts of body	0.0 (0.0-0.0)	0.0 (0.0-0.0)	0.022
Weight loss	0.0 (0.0-33.3)	0.0 (0.0-33.3)	0.224
Postoperative PROs (QLQ-C30), median (IQR)			
Physical functioning	86.7 (80.0-93.3)	86.7 (86.7-100.0)	0.103
Role functioning	83.3 (66.7-100.0)	100 (66.7-100.0)	0.323
Emotional functioning	100.0 (83.3-100.0)	95.8 (75.0-100.0)	0.462
Cognitive functioning	100.0 (83.3-100.0)	83.3 (83.3-100.0)	0.706
Social functioning	83.3 (66.7-100)	66.7 (83.3-100.0)	0.937
Global QOL	66.7 (50.0-83.3)	70.8 (52.1-83.3)	0.225
Fatigue	22.2 (11.1-33.3)	28.8 (2.8-33.3)	0.446
Nausea or vomiting	0.0 (0.0-0.0)	0.0 (0.0-0.0)	0.298
Pain	16.7 (0.0-25.0)	16.7 (0.0-33.3)	0.858
Dyspnoea	33.3 (33.3-33.3)	33.3 (8.3-33.3)	0.633
Sleep	0.0 (0.0-33.3)	0.0 (0.0-33.3)	0.933
Appetite loss	0.0 (0.0-0.0)	0.0 (0.0-0.0)	0.951
Constipation	0.0 (0.0-0.0)	0.0 (0.0-0.0)	0.278
Diarrhoea	0.0 (0.0-0.0)	0.0 (0.0-0.0)	0.369
FinanciaI difficulties	33.3 (0.0-33.3)	0.0 (33.3-33.3)	0.824
Postoperative PROs (QLQ-LC29), median (IQR)			
Coughing	16.7 (0.0-33.3)	16.7 (0.0-33.3)	0.668
Shortness of breath	22.3 (11.0-33.3)	22.3 (11.0-33.3)	0.270
Side effects of treatment	2.7 (0.0-11.0)	2.7 (0.0-11.0)	0.868
Fear of progression	33.3 (0.0-33.3)	33.3 (0.0-33.3)	0.707
Surgery-related problems	6.7 (0.0-20.0)	6.7 (0.0-20.0)	0.822
Coughing blood/Haemoptysis	33.3 (0.0-33.3)	16.7 (0.0-33.3)	0.956
Pain in chest	33.3 (0.0-33.3)	33.3 (0.0-33.3)	0.479
Pain in arm or shoulder	0.0 (0.0-33.3)	0.0 (0.0-33.3)	0.111
Pain in other parts of body	0.0 (0.0-33.3)	0.0 (0.0-33.3)	0.821
Weight loss	0.0 (0.0-0.0)	0.0 (0.0-0.0)	0.254

QOL: quality of life; PROs: patient-reported outcomes; QLQ-LC29: Quality of Life Questionnaire-Lung Cancer 29; QLQ-C30: Quality of Life Questionnaire-Core 30.

表4为两组内术前与术后3个月生活质量比较。单孔组内术后3个月患者的咳嗽（P=0.038）、气短（P<0.001）和咯血（P<0.001）较术前严重。多孔组内术后3个月患者的咳嗽（P=0.042）、气短（P<0.001）、害怕疾病进展（P<0.001）、咯血（P<0.001）和胸痛（P=0.007）较术前严重。

**表4:** 两组患者组内术前与术后3个月的生活质量

Item	U-VATS (n=89)		P		M-VATS (n=41)		P
	Preoperative	Postoperative			Preoperative	Postoperative	
COU, median (IQR)	16.7 (0.0-33.3)	33.3 (0.0-33.3)	0.038		16.7 (0.0-33.3)	16.7 (0.0-33.3)	0.042
DY, median (IQR)	11.1 (0.0-11.1)	22.3 (11.0-33.3)	<0.001		0.0 (0.0-11.1)	22.3 (11.0-33.3)	<0.001
SE, median (IQR)	5.6 (0.0-8.3)	5.7 (2.7-11.0)	0.262		5.6 (0.0-11.1)	2.7 (0.0-11.0)	0.687
FP, median (IQR)	33.3 (16.7-33.3)	33.3 (0.0-33.3)	0.061		33.3 (33.3-66.7)	33.3 (0.0-33.3)	<0.001
HA, median (IQR)	0.0 (0.0-0.0)	33.3 (0.0-33.3)	<0.001		0.0 (0.0-0.0)	16.7 (0.0-33.3)	<0.001
PC, median (IQR)	0.0 (0.0-33.3)	0.0 (0.0-33.3)	0.077		0.0 (0.0-0.0)	33.3 (0.0-33.3)	0.007
PA, median (IQR)	0.0 (0.0-33.3)	0.0 (0.0-33.3)	0.651		0.0 (0.0-33.3)	0.0 (0.0-33.3)	0.868
PO, median (IQR)	0.0 (0.0-33.3)	0.0 (0.0-33.3)	0.106		0.0 (0.0-0.0)	0.0 (0.0-33.3)	0.257
WL, median (IQR)	0.0 (0.0-33.3)	0.0 (0.0-0.0)	0.115		0.0 (0.0-33.3)	0.0 (0.0-0.0)	0.052

COU: coughing; DY: shortness of breath; SE: side effects of treatment; FP: fear of progression; HA: coughing blood/haemoptysis; PC: pain in chest; PA: pain in arm or shoulder; PO: pain in other parts of body; WL: weight loss.

### 2.3 传统结局指标

表5为传统结局指标的比较。两组患者住院时间、出血量及淋巴结清扫方式等差异无统计学意义。多孔组在手术时间（120.0 min vs 85.0 min, P=0.001）、术后住院时间（6.0 d vs 4.0 d, P=0.020）较单孔组更长，多孔组术后早期严重并发症（39.0% vs 10.1%, P=0.011）较单孔组更多。

**表5:** 传统临床结局

Variables	U-VATS (n=89)	M-VATS (n=41)	P
Operative time, min, median (IQR)	85.0 (65.0-115.0)	120.0 (100.0-175.0)	0.001
Operative bleed, mL, median (IQR)	50.0 (50.0-50.0)	100.0 (100.0-200.0)	0.128
Length of hospital stay, d, median (IQR)	7.0 (6.0-8.0)	10.0 (7.0-11.0)	0.212
Length of postoperative hospital stay, d, median (IQR)	4.0 (3.0-5.0)	6.0 (5.0-7.0)	0.020
Perioperative complication, Clavien-Dindo classification, n (%)			0.011
<Grade 2 or no	80 (89.9)	25 (61.0)	
≥Grade 2	9 (10.1)	16 (39.0)	
Lymphadenectomy, n (%)			0.055
Systematic lymph node dissection	36 (40.4)	24 (58.5)	
Selective lymph node sampling	53 (50.6)	17 (41.5)	
Number of lymph nodes dissection, median (IQR)	12.0 (7.0-16.0)	9.0 (7.0-13.0)	0.352

## 3 讨论

2011年Gonzalez等^[20]^首次报道了单孔胸腔镜肺叶切除术。近年来，单孔胸腔镜手术方式胸外科领域的应用逐渐成熟，有研究^[21]^发现单孔与多孔胸腔镜肺叶切除术具有相似的传统结局，但有研究^[22]^报道肺叶切除术后早期单孔胸腔镜具有更好的生活质量。与以往的研究^[23]^类似，我们观察到所有患者术后3个月的生活质量较术前有不同程度的下降，部分症状在术后3个月时并未恢复到术前水平。有研究^[24]^发现患者术后疼痛、咳嗽和气短等症状长时间持续存在，部分症状甚至会持续超过1年。这提示无论是单孔胸腔镜还是多孔胸腔镜肺叶切除术可能会对患者的生活质量造成一定的影响，即使经过3个月，患者的生活质量仍未恢复到术前的状态。

在本研究基线资料中单因素分析结果显示，接受多孔胸腔镜手术方式的患者与接受单孔胸腔镜手术方式的患者相比，前者更倾向放置2根引流管。然而引流管的数量可能会影响患者的生活质量，因此，本研究将这一潜在的混杂因素纳入回归模型中对研究结局进行了校正。与以往的研究^[25]^相似，我们发现单孔组患者在术后3个月的生活质量并不优于多孔组。但我们团队既往研究结果^[13]^发现，单孔组肺叶切除术后早期患者的重度症状比例较多孔组更少，功能更好。我们推测原因可能是：（1）不同测量工具的敏感性和特异性有差别，不同的量表所得到结果可能会有差异；（2）两组患者在术后3个月的症状和功能状态的差异逐渐恢复；（3）测量结局指标不同，我们团队在既往研究中比较单孔及多孔术后早期的生活质量采用的是重度症状的比例，本研究比较的是患者症状的平均分。本研究发现QLQ-LC29对于手术症状的评估在术后3个月时具有相似的结果。这与我们以往的认知不同，随着切口数量的增加，理论上我们会认为手术部位的疼痛和伤疤疼痛等症状在多孔组的患者中应更为严重。我们推测可能是因为QLQ-LC29条目仅含有4个等级，量表等级越多，敏感性越强^[26]^。此外，QLQ-LC29手术相关症状维度由5个条目组成，例如，量表中“您有手术部位疼痛吗?”这一条目的差异可能会被其他4个条目所抵消。QLQ-LC29能否用于评估肺癌外科患者的长期生活质量仍需高质量循证证据支持。

在传统结局指标中，单孔胸腔镜和多孔胸腔镜肺叶切除术均能达到根治的效果，两种手术方式均是有效、安全的。与以往的研究^[9]^相同，本研究显示单孔组在手术时间、出血量、住院时间、术后住院时间和术后并发症分级方面优于多孔组。多孔组术后严重并发症的发生率较高，我们推测原因可能是：（1）多孔组放置引流管更多，在术后早期造成患者的咳嗽和术后活动较差，从而增加肺部感染等并发症的发生率；（2）多孔组切口数量更多，切口数量增多可能提高切口愈合差、切口裂开和切口感染等并发症的发生率。本研究中单孔组手术时间比多孔组更短，可能的原因为：（1）多孔组清扫的淋巴结更多，需要花费更多的时间；（2）多孔组需要更多的时间用于切开、缝合额外的辅助操作切口。有研究^[27]^表明，手术时间的延长与术后并发症的概率增加有关，手术时间的减少可能会避免发生肺部感染、术后心律失常和持续性漏气等影响患者生活质量的严重并发症。此外，不同的主刀医师也可能是造成两组并发症具有差异的原因。单孔胸腔镜可能更有利于患者早期的症状及功能状态的恢复。

淋巴结清扫方式是患者预后的主要评价指标^[28]^，本研究中两组淋巴结清扫个数及清扫站数均无统计学差异，可能原因有以下几点：（1）单孔胸腔镜手术能够提供一种类似开放视角的方式，主刀医师对手术操作直接进行判断，提高了手术操作的准确度；（2）本研究入组的患者时间段集中在2021年，此时四川省肿瘤医院胸外科已常规开展单孔手术操作技术，主刀医师已经完成单孔操作的学习曲线。尽管单孔胸腔镜与多孔胸腔镜同样可达到肺癌根治术的淋巴结清扫要求，但仍需通过长期的随访来观察患者的预后。

本研究的主要局限性在于：（1）本研究为单中心研究，且样本量偏少，未来需要扩大研究中心和样本量，以验证结果的准确性；（2）本研究术后仅进行了单次PROs评估，可能并不能完全反映两组之间的生活质量差异和趋势；（3）本研究未能反映术中胸腔黏连状况及门钉淋巴结的处理方式；（4）本研究为观察性研究，存在固有混杂和偏倚，研究结论尚需随机对照研究来证明。

综上所述，本研究采用QLQ-LC29量表进行肺癌术后生活质量的测量，研究发现单孔胸腔镜肺叶切除术后3个月时患者的生活质量并不优于多孔胸腔镜肺叶切除术。单孔组在手术时间、术中出血量、术后住院时间及术后短期并发症等传统临床结局指标上可能具有一定的优势。该研究结论尚需要开展大样本、多中心随机对照研究来证明。

**Competing interests**

The authors declare that they have no competing interests.
